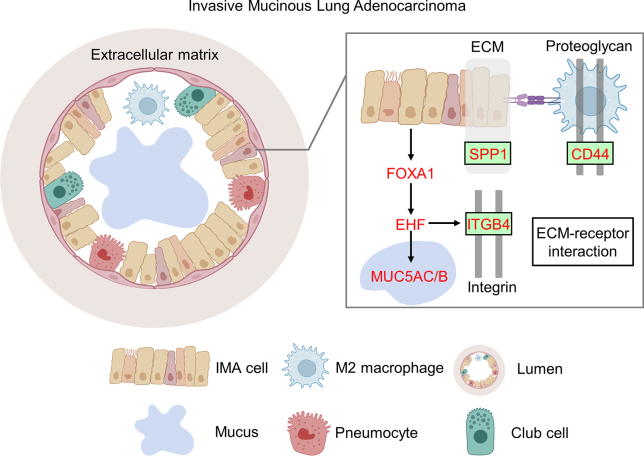# Transcriptional programs associated with luminal play a vital role in invasive mucinous lung adenocarcinoma

**DOI:** 10.1016/j.gendis.2024.101278

**Published:** 2024-03-26

**Authors:** Shufan Zhang, Rong Jiang, Changguo Wang, Manqiu Yang, Tao Wang, Jianzhou Cui, Guangbin Li, Shaomu Chen, Moli Huang

**Affiliations:** a School of Biology and Basic Medical Sciences, Soochow University, Suzhou, Jiangsu 215123, China; b Department of Pulmonary and Critical Care Medicine, The First Affiliated Hospital of Soochow University, Suzhou, Jiangsu 215006, China; c Cambridge-Suda Genomic Research Center, Soochow University, Suzhou, Jiangsu 215123, China; d Immunology Translational Research Programme, Yong Loo Lin School of Medicine, National University of Singapore, Singapore 117456, Singapore; e Immunology Programme, Life Science Institute, National University of Singapore, Singapore 117456, Singapore; f NUS-Cambridge Immunophenotyping Centre, National University of Singapore, Singapore 117456, Singapore; g Department of Thoracic Surgery, The First Affiliated Hospital of Soochow University, Suzhou, Jiangsu 215006, China

Invasive mucinous lung adenocarcinoma (IMA) is an aggressive subtype characterized by the presence of tumor cells with goblet cell morphology and abundant intracytoplasmic mucin. A previous study had shown that all epithelial tumors share similar gene expression-based luminal/basal subtypes and can impact treatment response.^1^ We identified the subtype of mucinous adenocarcinoma and found that many of them exhibit a luminal phenotype, particularly IMA. We established a single-cell atlas of the transition from lung adenocarcinoma (LUAD) to IMA and found that the luminal phenotype of IMA is characterized by high expression of *FOXA1* and specific enrichment of the extracellular matrix (ECM)-receptor interaction pathway. CellChat analysis revealed that the *SPP1-CD44* axis mediated communication between IMA and M2 macrophages. By chromatin immunoprecipitation sequencing analysis, we observed consistent enrichment of differential histone modifications at the ECM pathway. The luminal subtype marker *FOXA1* is central to the luminal-associated transcriptional programs and may bind to super-enhancer regions near *EHF* and promote its expression. Furthermore, *EHF* can bind to the transcription start site region of the prognostic risk factor *ITGB4* and promote its expression. Overall, the luminal-associated transcriptional programs (*FOXA1*-*EHF*-*ITGB4*) and its downstream ECM-receptor interaction pathway (*SPP1*, *CD44*, *ITGB4*) play a crucial role in IMA, influencing its immunity and tumor risk (Fig. 1A).

**Figure 1:**
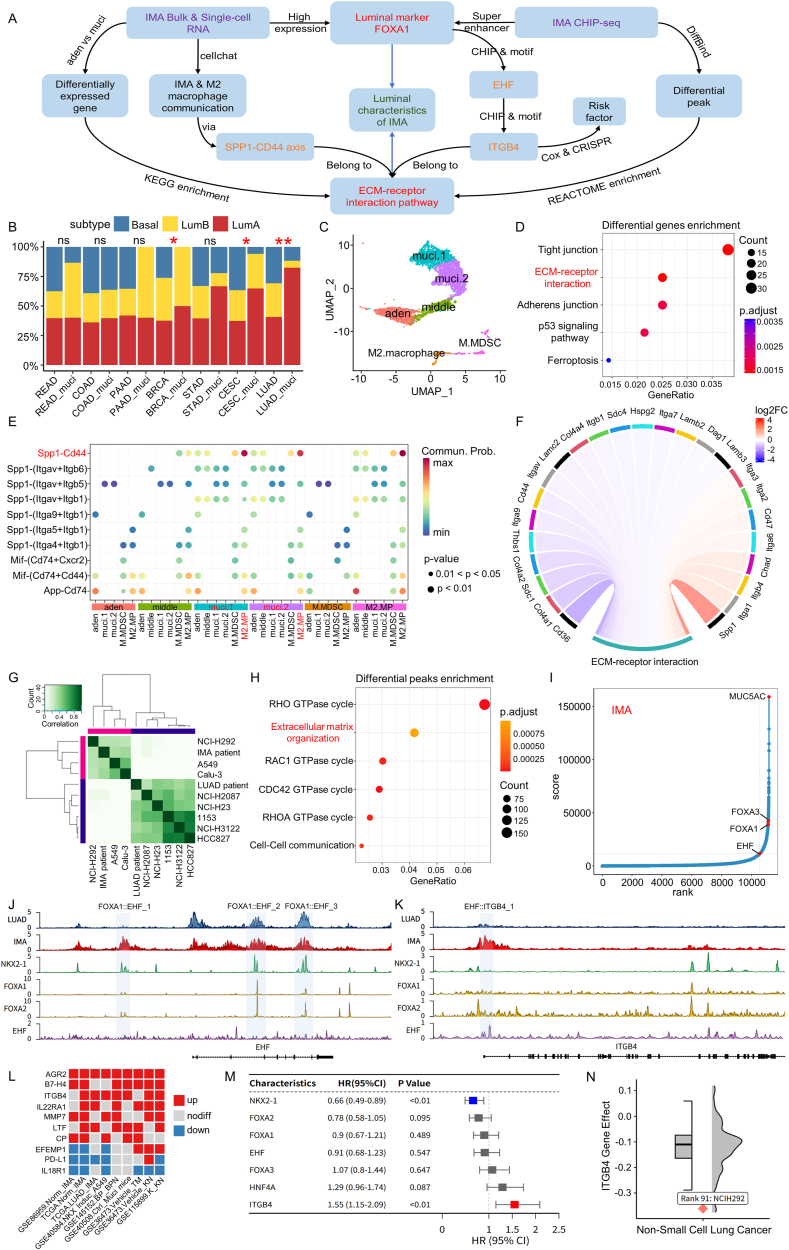
Integrated bulk & single-cell RNA and CHIP data reveal luminal-associated transcriptional programs in IMA and their roles. **(A)** An abstract circuit diagram. The aim is to analyze the luminal characteristics of IMA (green), which are mainly characterized by luminal marker *FOXA1* and ECM pathway (red). According to the data type, it is divided into two modules: RNA and CHIP (purple). **(B)** A stacked bar graph of the proportions of luminal and basal subtypes in adenocarcinoma and mucinous and Fisher's exact test was employed to detect differences in subtype proportions. **(C)** The UMAP plots showing annotated single-cell RNA sequencing profiles from *Nkx2-1* knockout LUAD mice, aden for LUAD, and muci for IMA. **(D)** The KEGG enrichment of differentially expressed genes (*P* < 0.05, |avg_log2FC| > 0.3) between aden cluster and muci clusters. **(E)** The heatmap of ligand-receptor cytokine pairs with a strong communication relationship between IMA cells and immune cells (macrophages abbreviated as MP). **(F)** The enrichment of genes showing significant differences between muci and aden clusters in the ECM-receptor interaction pathway (*P* < 0.05), with red color indicating up-regulation and blue color indicating down-regulation. **(G)** The DiffBind heatmap of differential peaks between four IMA and six LUAD tumor and cell lines. **(H)** REACTOME pathway enrichment analysis of differential peak genes annotated by ChIPseeker. **(I)** The hockey stick plots of an IMA patient showing rank-ordered H3K27ac signals for super-enhancer-associated genes. **(J, K)** Chromatin accessibility of *EHF* and *ITGB4*. Blue and red for H3K27ac CHIP of LUAD and IMA tumor samples, green and yellow for *NKX2-1*, *FOXA1*, and *FOXA2* CHIP of A549, and purple for *EHF* CHIP of Calu-3. The shaded areas are the *FOXA1/2* and *EHF* binding sites scanned by fimo, from EHF_1 to EHF_3 and ITGB4_1. **(L)** The expression of the top ten ligand-receptor genes in multiple IMA RNA sequencing datasets, where red represents significant up-regulation and blue represents significant down-regulation (*P* < 0.05, |log2 foldchange| > 1). **(M)** The survival risk forest plot of IMA-associated genes in the TCGA-LUAD cohort. Hazard ratio (HR) > 1 means increased survival risk. **(N)** CRISPR gene effect of *ITGB4* in NSCLC cell lines. IMA, invasive mucinous lung adenocarcinoma; CHIP, chromatin immunoprecipitation.

In our examination of TCGA patients with mucinous adenocarcinoma, we investigated the distribution of luminal A, luminal B, and basal-like tumors (Table S1). Our findings indicate that mucinous tumors exhibit a higher proportion of luminal subtypes, while mucinous LUAD (IMA) shows the highest proportion of luminal A subtype, exceeding 80% (Fig. 1B). Not coincidentally, we also observed high proportions of luminal A subtype in other IMA datasets (Fig. S1A). As mentioned in a previous study, the functional abnormalities in *NKX2-1* contribute to the formation of IMA.^2^ Our analysis revealed that low *NKX2-1* expression in IMA is primarily caused by the luminal subtype (Fig. S1B). The difference in gel-forming mucin *MUC5AC* between IMA and LUAD is primarily driven by the luminal A subtype (Fig. S1C). This suggests that mucin production in IMA may be associated with the luminal A subtype. The luminal marker *FOXA1* had fairly high expression in all four IMA datasets (Fig. S1D). Although the expression of *FOXA1* was lower in TCGA-IMA than in TCGA-LUAD, the expression level of its similar partner gene, *FOXA2*, was higher in IMA (Fig. S1E).

Due to incomplete recombination, single-cell RNA sequencing data from *Nkx2-1* knockout LUAD mice still retain *Nkx2-1* (Fig. S2A, B).^3^ According to marker genes, clusters 0–3 were identified as four major tumor clusters, and clusters 6 and 7 were annotated as human-like M-MDSC and M2 macrophages (Fig. S2C). Through pseudotime analysis and the expression level of *Nkx2-1* (Figs. S2D–F), we can infer that cluster 2 represents the initial adenocarcinoma cells, while clusters 0 and 1 are mucinous cells formed after *Nkx2-1* knockout, and cluster 3 represents an intermediate state of LUAD transitioning towards IMA (Fig. 1C). CytoTRACE scoring assessed the stemness levels of the four tumor clusters, further confirming this single-cell transition atlas (Fig. S2G, H). The luminal tendency of muci clusters was significantly higher than that of aden clusters (Fig. S2I). Differential analysis between aden and muci clusters revealed enrichment in the ECM-receptor interaction pathway (Fig. 1D).

Kim et al demonstrated that the density and morphology of ECM influence lumen formation, with ECM acting as a microenvironment and scaffold that regulates cell proliferation and survival through cell communication.^4^ Significant communications were observed between tumor cells and immune cells through three signaling pathways: *SPP1*, *MIF*, and *APP* (Figs. S3A–C). IMA cells displayed distinctive and robust communication with M2 macrophages, primarily mediated by the *Spp1*-*Cd44* axis (Fig. 1E). Additionally, we observed that middle, muci.1, and muci.2 cell clusters exhibited the same secreting cell patterns in their outgoing communication of *SPP1* signals (Fig. S3D). In the incoming communication patterns of target cell analysis, M-MDSC and M2 macrophage cell clusters belonged to the same pattern as recipients of *MIF* signals (Fig. S3E). Notably, within the ECM-receptor interaction pathway, *Spp1* and *Itgb4* were significantly up-regulated in IMA cells, with *Cd44* showing high expression in M2 macrophages (Fig. 1F; Fig. S3F).

Differential peaks in H3K27ac chromatin immunoprecipitation unveil significant epigenetic disparities between IMA and LUAD (Fig. 1G). These differential peaks demonstrate enrichment in pathways such as the RHO GTPase cycle, ECM, and cell–cell communication (Fig. 1H). In the IMA patient case, *FOXA1*, *FOXA3*, *EHF*, and *MUC5AC* were predicted to be super-enhancer-associated genes (Fig. 1I). These epigenetic features share remarkable similarities with the above transcriptomic results.

Utilizing the *Foxa1/2* chromatin immunoprecipitation sequencing from *Nkx2-1* negative and positive mice, we identified potential binding sites of *Foxa1/2* and conducted HOMER analysis to uncover enriched motifs within these regions. The results revealed that *Foxa3*, *Ehf*, and *AP-1* were enriched in *Nkx2-1* negative mice, while *Foxa3* and *Nkx2-1* were enriched in *Nkx2-1* positive mice (Fig. S4A). *NKX2-1* knockout and induction experiments revealed that the expression of *FOXA1*, *EHF*, and *MUC5AC/B* was all suppressed by *NKX2-1* (Fig. S4B, C and Table S2). Overexpression of *EHF* promoted the expression of *MUC5AC/B* and *SPDEF*, while si*EHF* down-regulated the expression of *SPDEF* (Fig. S4D, E). Notably, *EHF* did not impact the expression of *FOXA1*, indicating that *EHF* occupies a relatively downstream position in regulation. The chromatin accessibility of *EHF* was higher in IMA than in LUAD, while *FOXA1/2* shared proximal binding sites with *NKX2-1* (Fig. 1J and Table S3). This suggests that in the absence of *NKX2-1*, *FOXA1* may occupy its binding sites and contribute to the up-regulation of *EHF*, thereby promoting mucus expression.

Given the enrichment of cell communication and the recognition that ECM itself engages in signaling through integrin connections, we examined integrin genes in the ECM pathway and identified *EHF* binding to significantly differentially accessible regions in *ITGB4* chromatin (Fig. 1F–K and Table S3). The expression of ligand-receptor genes across multiple IMA datasets revealed that *AGR2* exhibited significant up-regulation in all IMA datasets, while the immune checkpoint *B7–H4* and the integrin *ITGB4* were significantly elevated in 78% of the datasets (Fig. 1L and Table S4). *ITGB4* emerged as a survival risk factor in the whole TCGA-LUAD cohort (Fig. 1M), while both RNA and protein levels of *ITGB4* were elevated in IMA (Fig. S4F, G). The impact of *ITGB4* knockout on the survival of the IMA cell line NCI–H292 surpassed that observed in NSCLC, underscoring *ITGB4*'s potential as a therapeutic target for IMA (Fig. 1N).

A study conducted by Maeda et al revealed that the positivity rate of *B7–H4* in IMA is significantly higher than that of *PD-L1*.^5^ TIDE analysis indicated a higher proportion in IMA of the response to immune checkpoint antibody treatments (Fig. S5A). Within two datasets, we observed a substantial enrichment of MDSC and M2 macrophage signatures in IMA (Fig. S5B, C). The correlations between *B7–H4*, MDSC scores, and M2 macrophage scores were significantly positive (Fig. S5D). The results unveiled the potential of *B7–H4* as an immunotherapy checkpoint in IMA, which may be associated with the two aforementioned immune cells.

In conclusion, our study reveals the luminal-associated transcriptional program (*FOXA1*-*EHF*-*ITGB4*) with *FOXA1* as the core regulator, as well as its downstream pathway (ECM-receptor interaction), which affects immune communication, mucus production, and tumor risk in IMA. Moreover, we identified *ITGB4* and *VTCN1* as potential therapeutic targets for IMA patients.

## Ethics declaration

The IMA tumor samples were collected from the First Affiliated Hospital of Soochow University. The experiments were conducted following the approved protocols by the Ethics Committee of the First Affiliated Hospital of Soochow University (No. 264, Batch 2021) and in accordance with the Helsinki Declaration.

## Author contributions

Shufan Zhang and Moli Huang designed the study, implemented the algorithm, and performed the analysis. Shufan Zhang, Rong Jiang, and Moli Huang wrote the manuscript. Changguo Wang, Manqiu Yang, Tao Wang, Jianzhou Cui, Guangbin Li, and Shaomu Chen helped collect the data and prepared the figures and tables. All authors read, reviewed, and approved the final manuscript.

## Conflict of interests

The authors declared no conflict of interests.

## Funding

This work was supported by the National Natural Science Foundation of China (No. 32370598, 31971117), the National Key R&D Program of China (No. 2018YFA0801100), the Natural Science Foundation of Suzhou, Jiangsu, China (No. SYS201517), and the Priority Academic Program Development of Jiangsu Higher Education Institutions (China).

## Data availability

H3K27ac chromatin immunoprecipitation sequencing data of the IMA patient and H292 cell line in this study are available under proper request (please contact huangml@suda.edu.cn). All other data can be accessed from public databases such as TCGA, CCLE and GEO.

## Figures and Tables

**figs1:**