# Supplementary material for: Transcriptional programs associated with luminal play a vital role in invasive mucinous lung adenocarcinoma
Source: Genes Dis. 2024 Mar 26;12(2):101278. doi: 10.1016/j.gendis.2024.101278 (PMC11582536; doi:10.1016/j.gendis.2024.101278)
Supplement: Multimedia component 1 [file mmc1.pdf]

Transcriptional programs associated with luminal play a vital role in invasive mucinous lung adenocarcinoma

Shufan Zhang [1], Rong Jiang [1], Changguo Wang [2], Manqiu Yang [1], Tao Wang [3], Jianzhou Cui [4,5,6], Guangbin Li [7], Shaomu Chen [7], Moli Huang [1,*]

Corresponding authors:

Moli Huang, School of Biology and Basic Medical Sciences, Soochow University, Suzhou 215123, China.

Telephone: +86 18013564796, E-mail: huangml@suda.edu.cn

This PDF file includes:

Materials and Methods

Figures S1-S5

Materials and Methods

Data acquisition

TCGA pan-cancer subtype analysis utilized a total of 2968 samples across 7 types of tumors, with RNAseq data obtained from UCSC Xena. CCLE expression profiles of 127 LUAD/IMA cell lines and CRISPR gene effect scores for 93 NSCLC cell lines were downloaded from the depmap portal. Single-cell RNA-seq was sourced from the GEO database, combining data from two mouse samples (GSM4307391 & GSM4307392).

H3K27ac CHIP-seq experiments involving the H292 cell line and an IMA patient were conducted by collaborative laboratories, and the samples were subsequently sent to Novogene and BGI Genomics for sequencing. The remaining 8 IMA/LUAD H3K27ac CHIP-seq datasets were also retrieved from the GEO database (A549: GSE91248, Calu-3: GSE63398, LUAD patient: GSM3732776, H2087: GSE182384, H23: GSE104481, 1153: GSE150975, H3122: GSE81482, HCC827: GSE76783). The sources of TF CHIP-seq data are as follows: *FOXA1/2* CHIPseq of *NKX2-1* positive/negative mouse lung adenocarcinoma (GSE43252); *NKX2-1*, *FOXA1*, *FOXA2* CHIP-seq of A549 cell line (GSE86957, GSM1010725, GSM1010724); *EHF* CHIP-seq of Calu-3 cell line (GSM1548072).

The induced and knockout expression profiles of *NKX2-1* in A549 and mouse were obtained from GSE40584 and GSE40508, respectively. The interference and overexpression RNA-seq data of *EHF* in Calu-3 were sourced from GSE63397. Additionally, several RNA-seq datasets comparing IMA with LUAD/Norm samples were included (6 IMA vs 6 Norm patient: GSE86959; 4 BPN vs 5 BP mice: GSE145152; 4 T-type vs 5 control, 5 KN-type vs 5 control: GSE36473; 3 KN vs 3 control: GSE115899). For detailed information about the datasets used in this study, please refer to Supplementary Table S5.

Luminal and basal subtyping

We used the original PAM50 algorithm for subtyping bulk samples into luminal and basal subtypes. The single-cell PAM50 signature is derived from scSubtype (https://github.com/Swarbricklab-code/BrCa_cell_atlas/tree/main/scSubtype).

Bulk RNA-seq analysis

The differential analysis for *NKX2-1* induction and knockout gene expression microarray data was computed using the Student's *t* test (GSE40584 & GSE40508). *EHF*-overexpressing and si*EHF* RNA-seq differential analysis were conducted with DESeq2 (GSE63397). Among multiple IMA-related datasets (Fig. 1L), genes were integrated based on the criteria of $P < 0.05$ and $|\log2FoldChange| > 1$ as the filtering threshold, including all significantly differentially expressed genes.

Single-cell RNA-seq analysis

Data merging and dimensionality reduction

Tumor single-cell RNAseq of two *Braf* $^{LSL\text{-}V600E/+}$; *Trp53* $^{f/f}$; *Nkx2-1* $^{f/f}$; *Rosa26* $^{LSL\text{-}tdTomato/LSL\text{-}tdTomato}$ mice were obtained from GSE145152. Single-cell expression data was analyzed using Seurat (4.3.0). Two BPN samples without any specific inhibitor treatment were selected for analysis (GSM4307391 & GSM4307392). Firstly, we merged the two tumor samples and filtered out cells with unique feature counts over 7500 or less than 200 and more than 20% mitochondrial counts. Then, normalized and scaled the data with default parameters. Finally, we performed UMAP dimensionality reduction clustering (20 dimensions) and used Harmony to remove batch effects.

Cell annotation

Referring to the data source used by the authors, filter out non-tumor cells based on the expression of well-known stromal markers (*Vim*, *Pecam1*, *Trpm5*, *Mgp*, *Ptprc*, *Cd79a*, *Cd3g*, *Itgam*, *Adgre1*, and Marco) and annotate tumor cells with tdTomato, *Epcam*, *Nkx2-1* and *Hnf4a* (Fig. S2C). Clusters 6 and 7 exhibit high expression of the immune marker gene Cd45 (*Ptprc*). Based on the expression of markers for alveolar macrophages (*Bhlhe41*, *Gpnmb*, *Trem2*, *Mrc1*), M1 macrophages (*Cd80*, *Cd86*, *Cd38*), and M2 macrophages (*Egr2*), Cluster 7 is annotated as M2 macrophages. Cluster 6 exhibits surface marker genes (*Cd33*, Cd11b/*Itgam*, *Cd14*) associated with human monocytic myeloid-derived suppressor cells (M-MDSC) and shows a similar expression pattern to the marker genes (*Cd14*, *S100a9*, Cd15/*Fut4*^{low}) found in MDSCs of non-small cell lung cancer (NSCLC) patients. Therefore, it is annotated as human-like M-MDSC.

Differential gene expression

Differential genes were calculated using the FindMarkers function from the Seurat package with default setting.

Tumor stemness and pseudotime analysis

Tumor stemness was calculated using CytoTRACE (version 0.3.3). Pseudotime analysis was conducted using Monocle 2, and the parameters were all referenced from the documentation (http://cole-trapnell-lab.github.io/monocle-release/docs/).

Cell Communication Analysis

The communication between cells and intercellular signaling were predicted using CellChat with default setting.

ChIP-seq experiment and analysis

The NCI-H292 cell line was generously provided by Dr. Fengying Wu laboratory at Shanghai Pulmonary Hospital. IMA tumor tissues were fresh specimens collected after surgery. For CHIP of tumor cells, we used 1% methanol to crosslink DNA to proteins and fragment chromatin with the Bioruptor Plus sonication device. ChIP was performed as previously described.

in brief, after sonication, cell debris was removed by centrifugation (14,000 × g, 10 min). The supernatant was preincubated with Dynabeads (Invitrogen) with H3K27ac antibody. After incubation overnight in the cold room, magnetic beads were washed from cold low salt wash buffer, high salt wash buffer, LiCl wash buffer to TE buffer. ChIP DNA was eluted, reverse-crosslinked, purified by QIAGEN QIAquick PCR purification kit. CHIP-enriched DNA and input DNA was used for high-throughput DNA sequencing. The NCI-H292 cell line and IMA patient tumor cells were sequenced using the NovaSeq 6000 and DNBSEQ platforms, respectively.

Genome alignment of CHIP-Seq data was done using Bowtie2 and the blacklist region was removed with bedtools (https://github.com/Boyle-Lab/Blacklist). Peaks were called using MACS2 with default parameters. Super-enhancers were computed with ROSE. The *FOXA1/2* ChIP-seq data from GSE43252 was analyzed using the findMotifsGenome.pl script from Homer to identify enriched motifs (Fig. S4A). Differential peaks of H3K27ac ChIP-seq between IMA and LUAD samples were computed using DiffBind, and the corresponding genes were annotated using ChIPseeker. Visualization of CHIP-seq peaks was performed using karyoploteR. The reference genomes utilized in this study include mm10 and hg19.

Gene set enrichment analysis

In IMA single-cell analysis, genes that are significantly upregulated in the muci cluster compared to the aden cluster were used as a signature for enrichment (*P* <0.05 & avg_log2FC >0.3). The clusterProfiler package was utilized to perform KEGG enrichment analysis (*P* <0.05). After annotating the differential peaks of H3K27ac between IMA and LUAD samples with ChIPseeker, we conducted REACTOME pathway enrichment analysis with ReactomePA package.

COX survival analysis and gene effect analysis

Batch univariate Cox analysis was performed using the survival package on the IMA-associated gene expression in the whole TCGA-LUAD dataset (Fig. 1M). The gene effect scores represent the impact of CRISPR gene knockout on cell line survival (Fig. 1N).

Statistical analysis

The statistical significance of all figures is denoted with symbols ("***", "**", "*", and "ns", corresponding to *P* values of 0-0.001, 0.001-0.01, 0.01-0.05, and 0.05-1). If not explicitly indicated, the default statistical test used is the Wilcoxon test.

Supplementary Figures

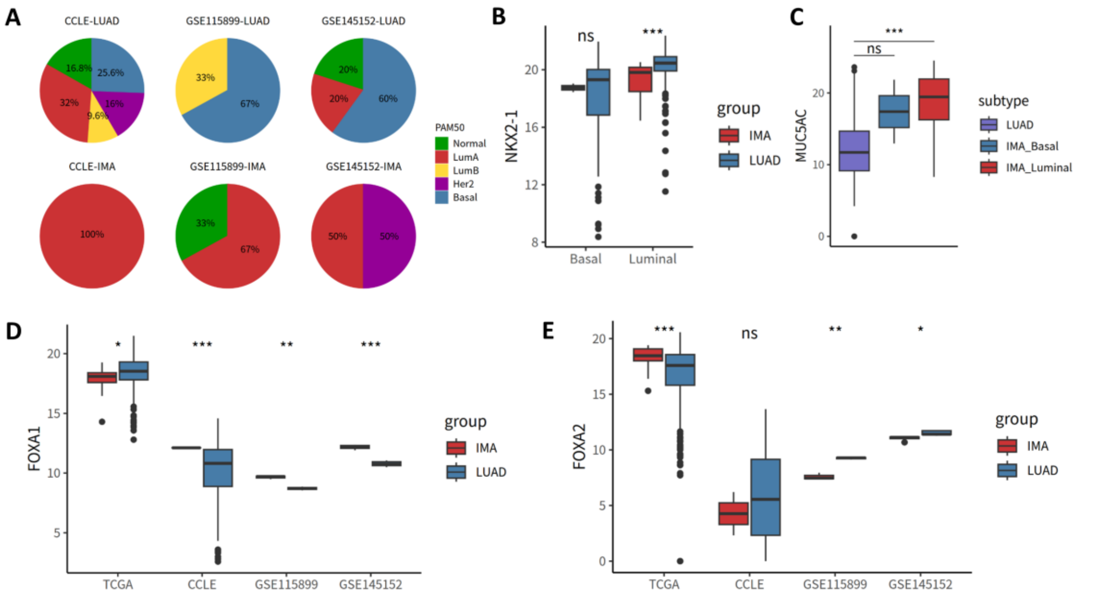

Supplementary Figure 1. Percentage of luminal subtypes in IMA and expression characteristics. (A) The PAM50 subtyping of LUAD and IMA in three datasets. (B-C) Gene expression boxplot of *NKX2-1* and *MUC5AC* in IMA and LUAD. (D-E) Boxplots of *FOXA1/2* expression in multiple datasets (Student's *t* test).

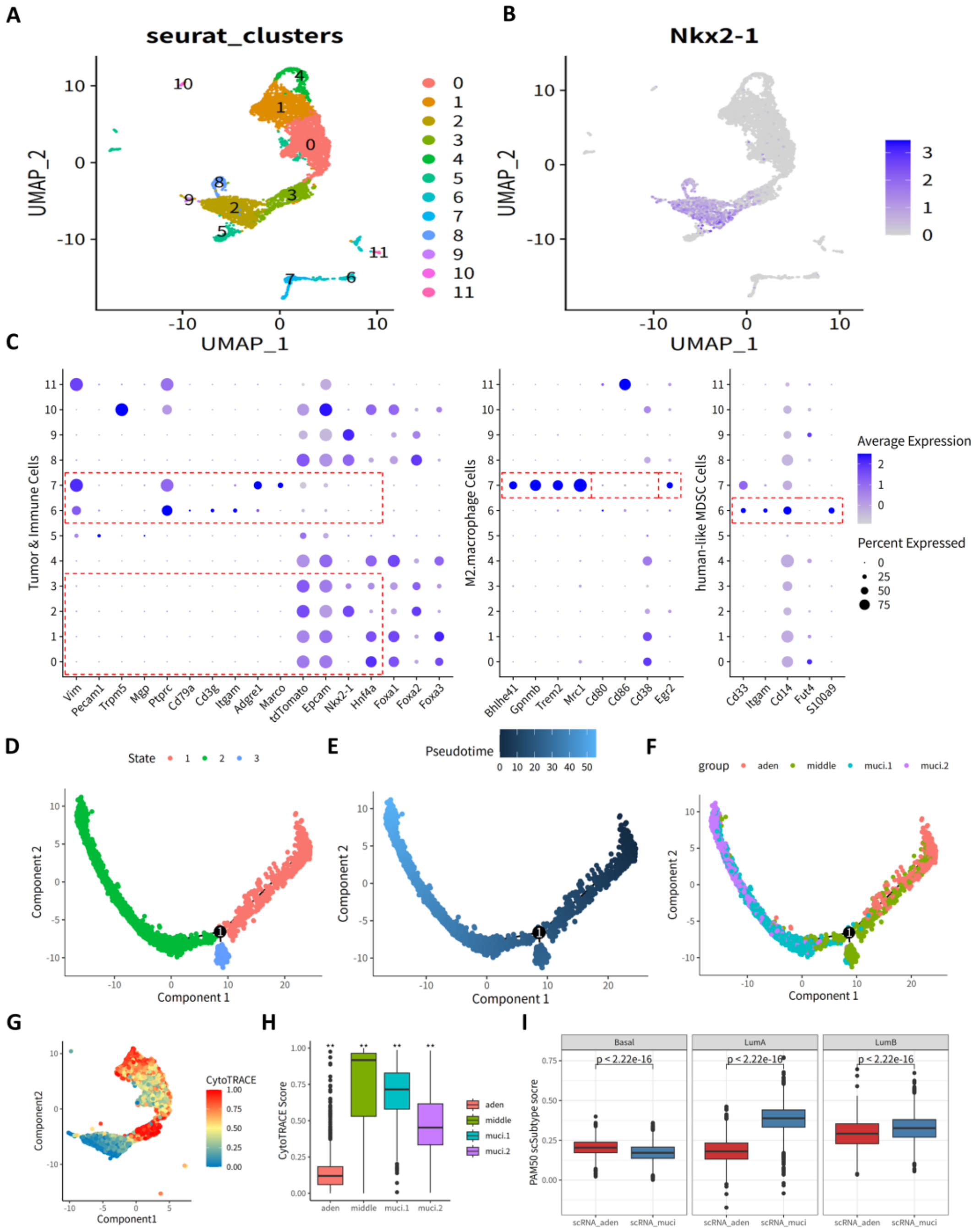

Supplementary Figure 2. Annotation and analysis of IMA single-cell atlas. (A) UMAP plots of merged single cells from 2 *Nkx2-1* knockout LUAD mice. (B) UMAP plot illustrating the expression levels of *Nkx2-1* in single cells. (C) Gene signature expression in single-cell data, annotated from left to right as follows: tumor cells (clusters 0-3), M2 macrophages (cluster 7), human-like M-MDSC (cluster 6). (D-F) Monocle 2 single-cell pseudo-temporal analysis revealed three stages of tumor cell development, progressing from right to left. The aden cluster represented the initial stage, the middle cluster represented the intermediate stage, and the muci.1 and muci.2 clusters represented the final stage. (G-H) Heatmap and boxplot of CytoTRACE scores for tumor cell clusters, which correspond to transcriptional diversity. Differentiated cells have low transcriptional diversity and low scores, while proliferating cells have high scores. (I) PAM50 subtype enrichment scores of aden and muci.1 & muci.2 in Single-cell RNA-seq.

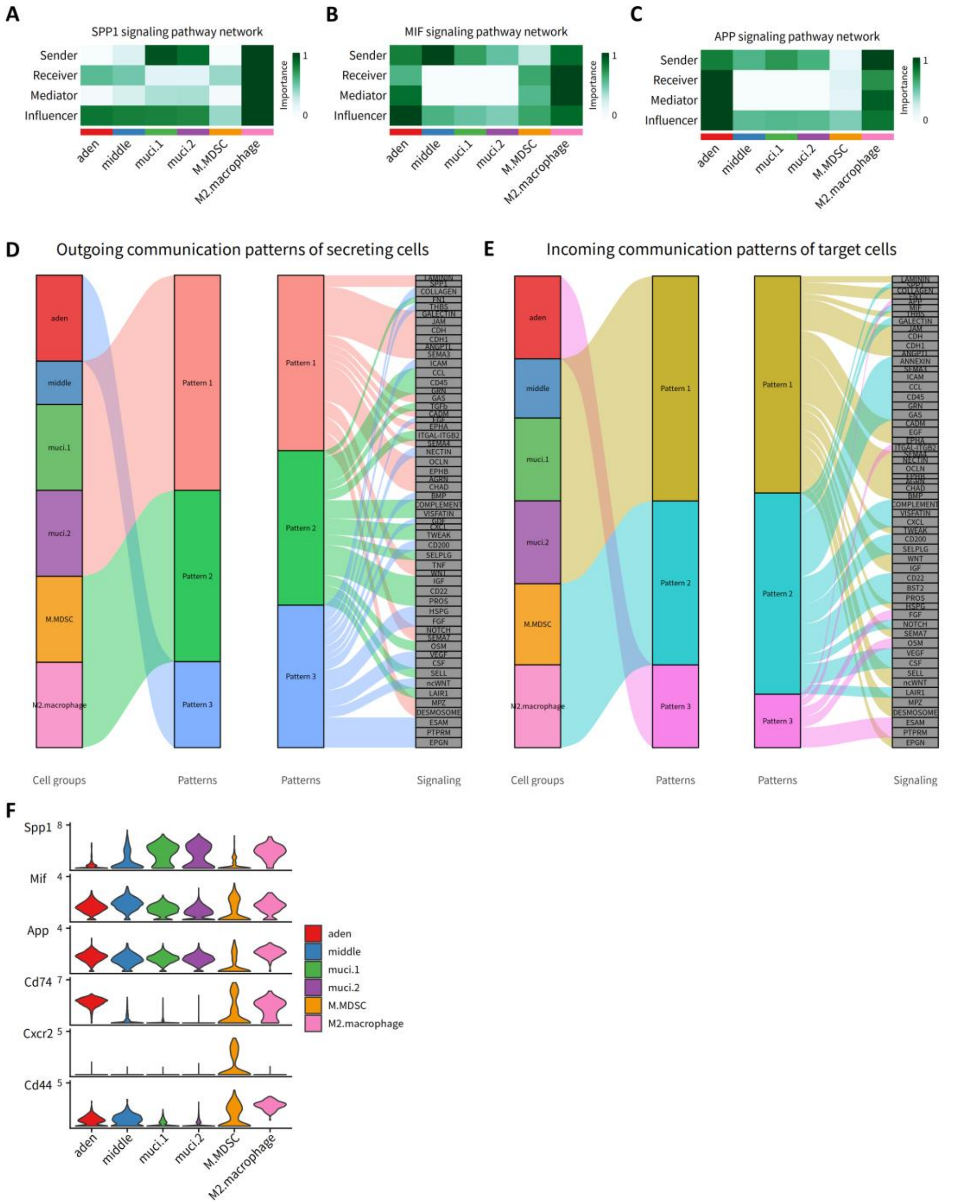

Supplementary Figure 3. Cell communication of IMA single-cell atlas. (A-C) Signaling roles of cell groups as well as the major contributing signaling of *SPP1*, *MIF*, *APP*. (D-E) River plot of outgoing communication patterns of secreting cells and incoming communication patterns of target cells. (F) Expression levels of ligand and receptor genes involved in cell communication related to *SPP1*, *MIF*, and *APP*.

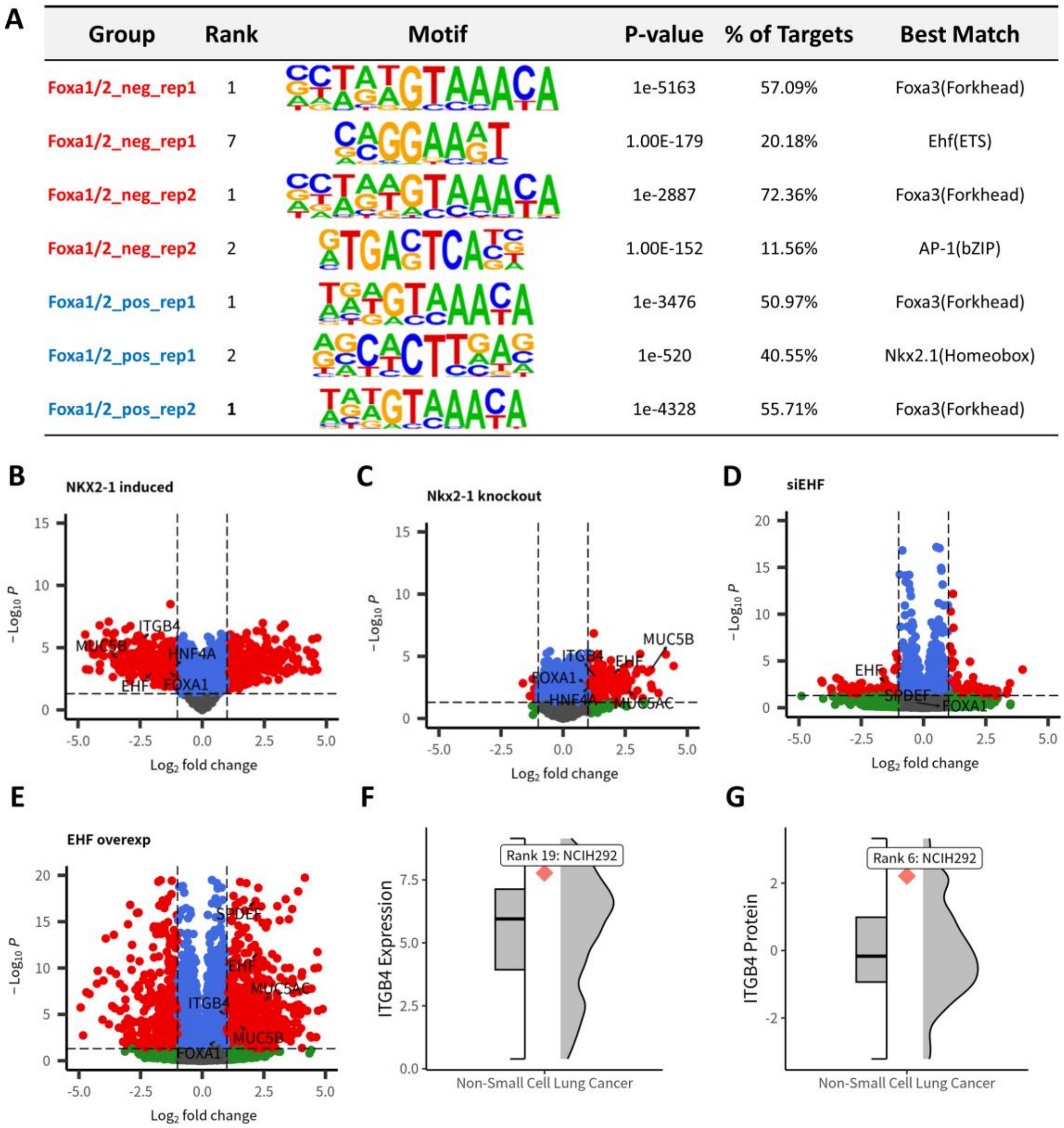

Supplementary Figure 4. Downstream regulation of luminal-associated genes. (A) Homer motif enrichment analysis of *Foxa1/2* CHIP-seq, with the red color representing the *Nkx2-1*-negative group, and the blue color representing the *Nkx2-1*-positive group. (B-C) Induction and knockout of *NKX2-1* in A549 and IMA mice. The horizontal and vertical dotted lines represent P=0.05 and log2FC=±1. (D-E) Overexpression and silencing of *EHF* in A549 and Calu-3 cell lines. (F-G) RNA and protein expression levels of ITGB4 in NSCLC cell lines.

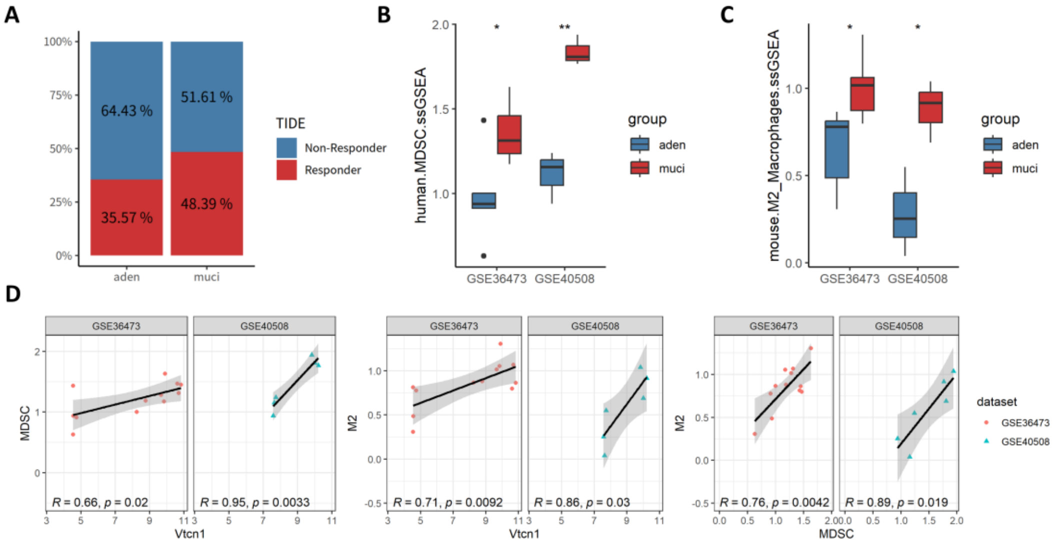

Supplementary Figure 5. Immunological study of B7-H4 in IMA. (A) The immune therapy response of LUAD and IMA patients in TCGA predicted by TIDE. (B-C) The ssGSEA enrichment scores of human-like MDSC and mouse M2 macrophages. (D) The pearson correlation between the RNA expression of B7-H4 (Vtcn1), MDSC enrichment scores, and M2 macrophage enrichment scores in the GSE36473 and GSE40508 datasets.
