# Supplementary material for: Transcriptional programs associated with luminal play a vital role in invasive mucinous lung adenocarcinoma
Source: Genes Dis. 2024 Mar 26;12(2):101278. doi: 10.1016/j.gendis.2024.101278 (PMC11582536; doi:10.1016/j.gendis.2024.101278)
Supplement: Multimedia component 3 [file mmc3.docx]

**Transcriptional programs associated with luminal play a vital role in invasive mucinous lung adenocarcinoma**

Cancer cells undergo lineage switching in the course of their natural history and in response to the selective pressure of targeted therapy. The loss of *NKX2-1* in human and murine lung adenocarcinoma leads to invasive mucinous adenocarcinoma (IMA), a lung cancer subtype that exhibits gastric differentiation and harbors a distinct spectrum of driver oncogenes^1^. IMA frequently carries "undruggable" *KRAS* mutations (63 to 90%) and exhibit a low expression of PD-L1, thus unable to benefit from *EGFR* inhibitors and Immune checkpoint blockade therapies.^2^ Therefore, IMA urgently needs new oncogene targets and immune checkpoint targets to improve its treatment.

A previous study had shown that all epithelial tumors share similar gene expression-based luminal/basal subtypes and can impact treatment response.^3^ *FOXA1* is a marker of luminal subtype in breast cancer and is associated with its survival.^4^ Meanwhile, *FOXA1* and *FOXA2* are required to initiate mucinous *NKX2-1*-negative lung adenocarcinomas and activate their gastric differentiation program.^5^ Therefore, we took FOXA1 as a starting point to investigate the luminal-associated transcriptional program in order to identify new potential molecular targets for IMA.

PAM50 algorithm were employed to examine the proportions of Basal, LumA, and LumB subtypes in seven types of mucinous adenocarcinoma. The results showed a significant increase in the proportion of luminal subtypes in mucinous BRCA, mucinous CESC and IMA. Subtype scoring of single-cell data from IMA mice also demonstrated a significant luminal-enrichment tendency. Integrated analysis of RNA-seq and ChIP-seq data revealed that *FOXA1* exhibits a high expression level and high chromatin accessibility in IMA. These results provide preliminary evidence of the luminal tendency of IMA and the active status of *FOXA1*.

We used Homer to perform de novo motif discovery on Foxa1/2 CHIP-seq data and check the enrichment of known motifs. In *Nkx2-1*-positive LUAD mouse, there was a significant enrichment of *Nkx2-1* and *Foxa3* motifs, while *Ehf*, *Foxa3*, and *AP-1* motifs were enriched in *Nkx2-1*-negative IMA mouse. Through the analysis of H3K27ac and TF CHIP-seq data from many IMA and LUAD samples, we found that *FOXA1* may bind to super-enhancer regions near *EHF* and promote its expression. Meanwhile, Overexpression of *EHF* promotes the expression of mucin-related genes *MUC5AC/B* and *SPDEF*, while siEHF downregulates the expression of *SPDEF*. These outcomes suggest that in the absence of *NKX2-1*, *EHF* replaces *NKX2-1* as a partner for *FOXA1*, potentially promoting the mucinous program in IMA.

The differentially expressed genes between IMA and LUAD cells are specifically enriched in the ECM-receptor interaction pathway. Simultaneously, the differential H3K27ac peaks between IMA and LUAD samples are enriched in extracellular matrix organization pathway. Notably, our single-cell communication study revealed that ECM-receptor interaction pathway genes *SPP1* and *CD44* mediate communication between IMA and M2 macrophages. Furthermore, *EHF* was found to bind to the TSS region of *ITGB4* and promote its expression. *ITGB4* is also a gene in the ECM-receptor interaction pathway, and COX regression survival analysis indicates that it serves as a prognostic risk factor in IMA. In summary, the luminal- associated transcriptional regulatory programs, centered around *FOXA1*, extends to *EHF* and *ITGB4*, with the ECM-receptor interaction pathway serving as a downstream pathway.

In order to identify new oncogene targets and immune checkpoint targets in IMA, we compared the expression of ligand/receptor genes with significant changes in all nine IMA datasets. *AGR2* was significantly upregulated in all IMA datasets, while *ITGB4* and *VTCN1* (B7-H4) were significantly upregulated in 7/9 datasets. It has been reported in previous years that AGR2 is activated by FOXM1 and leads to the progression of LUAD to IMA.^6^ To evaluate the potential of *ITGB4* as an oncogene target in IMA, we compared the CRISPR gene effect scores of IMA cell line NCI-H292 with other SCLC cell lines. The results showed that ITGB4 knockout significantly affected the survival of NCI-H292 cell line. Recently, a study by Maeda et al. revealed that the positivity rate of B7-H4 (64%) in IMA is significantly higher than that of PD-L1 (8%).^7^ This is consistent with our statistical results, but the specific relationships between immune checkpoint *VTCN1* and immune cells have not been studied. In the GSE36473 and GSE40508 datasets, we observed that IMA had significantly higher M-MDSC and M2 macrophage scores than LUAD. After calculating the correlation between the expression of VTCN1 and the scores of M-MDSC and M2 macrophages, we found that all three were significantly positively correlated. So far, we have identified the potential oncogene target *ITGB4* and immune checkpoint target *VTCN1* for IMA, and have made preliminary investigations into the immune cell types associated with *VTCN1*.

The latest research by David A. Tuveson et al. published in Gut suggests that a mucus production program promotes classical pancreatic ductal adenocarcinoma, and inactivation of the mucus regulator gene *SPDEF* leads to tumor differentiation from the classical to the basal-like subtype.^8^ This study provides a new horizons that, the mucus-associated genes may also play a role in maintaining the tumor subtype in mucin-producing tumors. In our study, *EHF* is a member of the luminal-associated transcriptional program, and its overexpression promotes the expression of mucin genes *MUC5AC/B* and *SPDEF* in IMA. In other words, *EHF* is associated with both luminal subtype and mucus functions in IMA. Therefore, future research on other mucin-producing tumors can also explore the relationship between specific subtypes and mucin programs.

In conclusion, our study reveals the luminal-associated transcriptional program (*FOXA1*-*EHF*-*ITGB4*) with *FOXA1* as the core regulator, as well as its downstream pathway (ECM-receptor interaction), which affect immune communication, mucus production, and tumor risk in IMA. Moreover, we identified *ITGB4* and *VTCN1* as potential therapeutic targets for IMA patients. More importantly, our findings resonate with the research of David A. Tuveson et al., proposing a new research direction for mucinous adenocarcinoma: programs for maintaining specific subtype of mucinous adenocarcinoma overlaps with its mucin production programs.


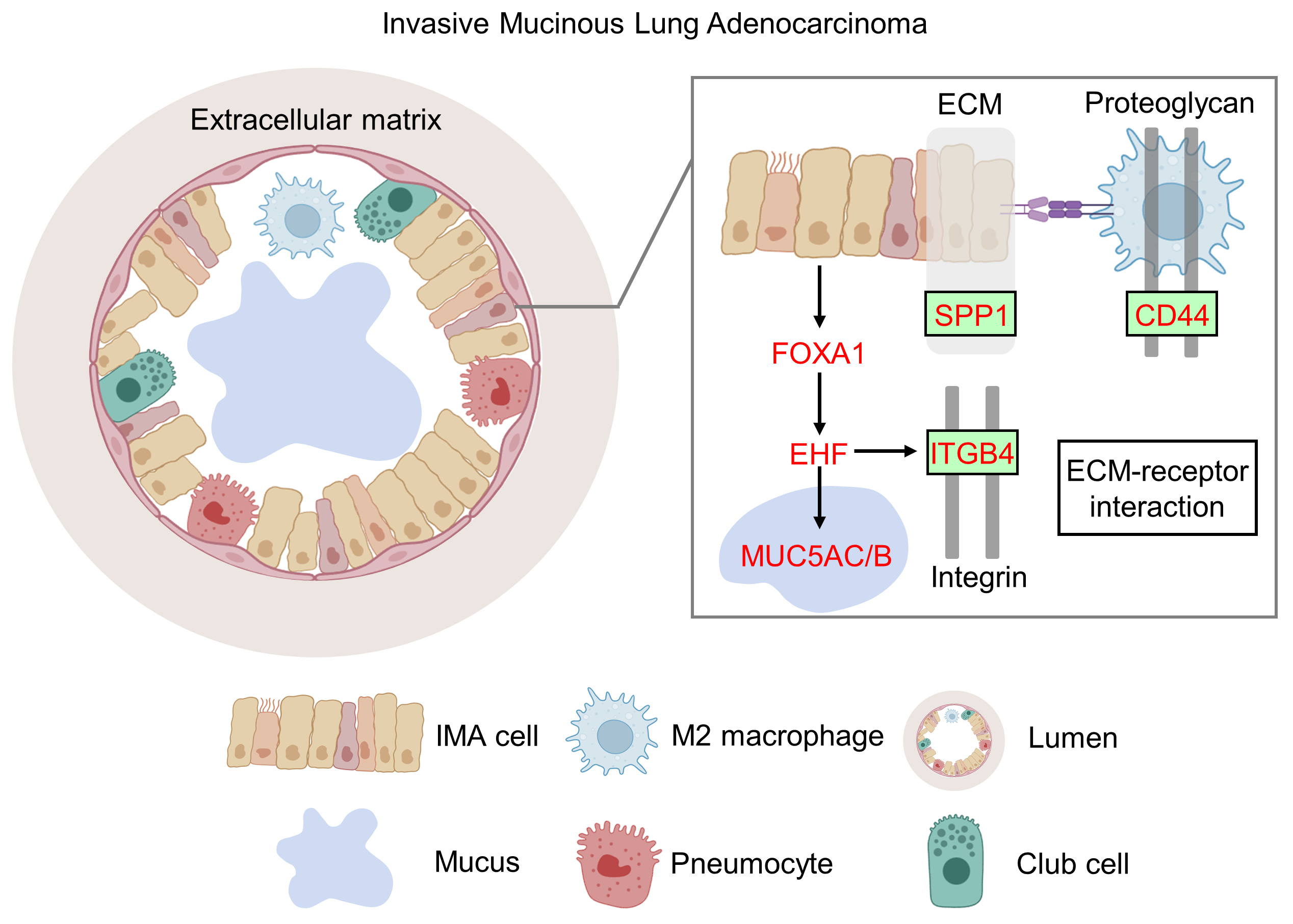


**In brief**

The luminal-associated transcriptional program (*FOXA1*-*EHF*-*ITGB4*) and downstream pathway (ECM-receptor interaction) affect the immune communication, mucus production, and tumor risk in IMA.

**Reference:**

1. Zewdu R, Mehrabad EM, Ingram K, et al. An NKX2-1/ERK/WNT feedback loop modulates gastric identity and response to targeted therapy in lung adenocarcinoma. Elife. Apr 6 2021;10

2. Xu L, Li C, Lu H. Invasive mucinous adenocarcinoma of the lung. Transl Cancer Res. Dec 2019;8(8):2924-2932.

3. Zhao SG, Chen WS, Das R, et al. Clinical and Genomic Implications of Luminal and Basal Subtypes Across Carcinomas. Clin Cancer Res. Apr 15 2019;25(8):2450-2457.

4. Metovic J, Borella F, D'Alonzo M, et al. FOXA1 in Breast Cancer: A Luminal Marker with Promising Prognostic and Predictive Impact. Cancers (Basel). Sep 27 2022;14(19)

5. Camolotto SA, Pattabiraman S, Mosbruger TL, et al. FoxA1 and FoxA2 drive gastric differentiation and suppress squamous identity in NKX2-1-negative lung cancer. Elife. Nov 26 2018;7

6. Milewski D, Balli D, Ustiyan V, et al. FOXM1 activates AGR2 and causes progression of lung adenomas into invasive mucinous adenocarcinomas. PLoS Genet. Dec 2017;13(12):e1007097.

7. Guo M, Tomoshige K, Meister M, et al. Gene signature driving invasive mucinous adenocarcinoma of the lung. EMBO Mol Med. Apr 2017;9(4):462-481.

8. Tonelli C, Yordanov GN, Hao Y, et al. A mucus production programme promotes classical pancreatic ductal adenocarcinoma. Gut. Jan 23 2024;
